# Comparison of Quantitative and Qualitative (Q)SAR Models Created for the Prediction of K_i_ and IC_50_ Values of Antitarget Inhibitors

**DOI:** 10.3389/fphar.2018.01136

**Published:** 2018-10-10

**Authors:** Alexey A. Lagunin, Maria A. Romanova, Anton D. Zadorozhny, Natalia S. Kurilenko, Boris V. Shilov, Pavel V. Pogodin, Sergey M. Ivanov, Dmitry A. Filimonov, Vladimir V. Poroikov

**Affiliations:** ^1^Department of Bioinformatics, Institute of Biomedical Chemistry, Moscow, Russia; ^2^Department of Bioinformatics, Pirogov Russian National Research Medical University, Moscow, Russia

**Keywords:** QSAR, antitarget, inhibition, adverse drug reactions, K_i_, IC_50_, GUSAR, ChEMBL

## Abstract

Estimation of interaction of drug-like compounds with antitargets is important for the assessment of possible toxic effects during drug development. Publicly available online databases provide data on the experimental results of chemical interactions with antitargets, which can be used for the creation of (Q)SAR models. The structures and experimental K_i_ and IC_50_ values for compounds tested on the inhibition of 30 antitargets from the ChEMBL 20 database were used. Data sets with K_i_ and IC_50_ values including more than 100 compounds were created for each antitarget. The (Q)SAR models were created by GUSAR software using quantitative neighborhoods of atoms (QNA), multilevel neighborhoods of atoms (MNA) descriptors, and self-consistent regression. The accuracy of (Q)SAR models was validated by the fivefold cross-validation procedure. The balanced accuracy was higher for qualitative SAR models (0.80 and 0.81 for K_i_ and IC_50_ values, respectively) than for quantitative QSAR models (0.73 and 0.76 for K_i_ and IC_50_ values, respectively). In most cases, sensitivity was higher for SAR models than for QSAR models, but specificity was higher for QSAR models. The mean *R*^2^ and RMSE were 0.64 and 0.77 for K_i_ values and 0.59 and 0.73 for IC_50_ values, respectively. The number of compounds falling within the applicability domain was higher for SAR models than for the test sets.

## Introduction

Adverse drug reactions (ADRs) are one of the main problems in drug discovery and clinical practice (Böhm and Cascorbi, 2016). According to some estimates, ADR is one of the leading causes of hospitalization and death in developed countries (Starfield, 2000; Kochanek et al., 2016), the second most common cause of drug attrition in later stages of clinical trials and the major reason for drug withdrawal from the market (Hornberg et al., 2014). This situation is largely due to disadvantages of traditional animal toxicological experiments and clinical trials that cannot detect all serious ADRs because of inter-species differences and their idiosyncratic nature. Therefore, additional methods including *in vitro* and *in silico* approaches are currently being developed. *In silico* approaches are usually based on machine learning techniques and network analyses to link several chemical and biological features of approved and withdrawn drugs to ADRs, which include molecular descriptors, known or predicted drug targets, drug-induced gene expression profiles and cell phenotypic features (Ivanov et al., 2016). These approaches allow predict dangerous ADRs in the early stages of drug development and provide insights into potential toxic mechanisms of drug candidates. It is currently accepted that the most ADRs are the consequence of unintended interactions of drugs with human protein targets and are not related to a therapeutic mechanism of action. For example, blocking HERG potassium channels in the heart causes life-threatening arrhythmias (Siramshetty et al., 2016). There are dozens of human proteins that have known relationships to ADRs, and corresponding information has accumulated in public databases (Ji et al., 2003; Zhang et al., 2007) and been described in some publications (Whitebread et al., 2005; Bowes et al., 2012). These proteins are called “antitargets” because to avoid dangerous ADRs, they should not interact with drugs. Many pharmaceutical companies use *in vitro* assays to measure interactions of lead compounds with “antitargets” and select the least promiscuous ones for further development. To avoid performing hundreds of experiments, such interactions can also be predicted using ligand-based structure-activity relationship analysis or docking (Ivanov et al., 2016; Simões et al., 2018). Due to accumulation of data on chemical-protein interactions and three-dimensional protein structures in public databases such as ChEMBL (Gaulton et al., 2017), PubChem (Wang et al., 2017), and PDB (Berman et al., 2000), it has become possible to predict interactions with many hundreds of human proteins, including “antitargets." There are plenty of published (Q)SAR models (Poroikov et al., 2007; Filz et al., 2008; García-Sosa and Maran, 2014; Ivanov et al., 2016) and free available web-services (Zakharov et al., 2012; Braga et al., 2015) that may perform such predictions; however, no study was found with a comparison between the accuracy of classification (SAR) and quantitative (QSAR) models created based on the same data, descriptors and mathematical algorithm. The aim of this work is the creation, validation, and accuracy estimation of SAR and QSAR models for the prediction of the inhibition of 30 antitargets using GUSAR software and data on structures and K_i_ and IC_50_ values of tested compounds from the ChEMBL 20 database. Earlier, we published a study on the creation of reasonable QSAR models by GUSAR software and the appropriate web service^1^ for the prediction of interaction between drug-like compounds and 18 antitargets (Zakharov et al., 2012). In this paper, we have significantly expanded the list of covered “antitargets" and significantly increased the volumes and diversity of training samples, which allowed us to expand the range of applicability of models and to obtain valuable results.

## Materials and Methods

### Data Sets

Structures and experimental K_i_ and IC_50_ values of compounds tested on the inhibition of 30 antitargets were extracted from the ChEMBL 20 database. The data sets with K_i_ and IC_50_ values including more than 100 compounds were created for each antitarget (**Table 1**). Only the records with K_i_ or IC_50_ values in nM and symbol “ = ” in the field “Relation” were extracted from ChEMBL database. During the creation of data sets of compounds interacting with receptors, we included records with compounds studied as truly antagonists and records with compounds studied on biding affinity because of we could not divided them. In spite of Ki and IC50 values indicate the affinity of a compound by a given receptor, and they do not necessarily provide functional information related with agonism or antagonism of a compound to such target we decided to include such data because antagonism of receptors may be related with Ki and IC50 values, whereas agonism to receptors are usually represented by EC50 values. K_i_ or IC_50_ values were transformed in pIC_50_ = −log10(IC_50_(M)) and pK_i_ = −log10(K_i_(M)) values. **Table 1** also shows the known relations between the inhibition of antitargets and ADRs. The number of compounds with K_i_ values was approximately 1.5 times higher than that for IC_50_ values (46830 and 29678, respectively). The sets included structures of single electroneutral small (molecular weight in range from 50 to 1250 Da) organic molecules. In general, such representation of structure corresponds to the best QSAR practice (Fourches et al., 2016) implemented in the GUSAR software, which was used in our study (see below). If a compound had several experimental values for the parameter, then a median value was used. Such median values were calculated because the reference compounds usually had several experimental values, since they were tested in many experiments. Deleting such compounds reduces an important part of chemical space and significantly restricts the applicability domain of the global QSAR models. In several publications related to the creation of global QSAR models based on heterogeneous data, authors used average values (Politi et al., 2014; Cortes-Ciriano and Bender, 2015). The median value was used because it better characterizes the set of values for strongly skewed distributions. Zip file including SD files related with the appropriate target (the gene name of targets is used in a file name), and endpoint is provided in **Supplementary Materials**. Each SD file includes structures, ChEMBL_ID, and experimental values. For classification models and comparison of prediction results between the SAR and QSAR models, 1 μM was used as a threshold between active and inactive compounds. The sets were sorted by the ascending mode of the appropriate values. Then, successively, a number from 1 to 5 was assigned for each structure from a set. After that, the sets were divided into five unique parts according to the assigned number of structures. These parts were used for the fivefold cross-validation (fivefold CV) procedure, when each unique part was used as an external test set, and the remaining parts were used as a training set. As a result, different five training and five external test sets for K_i_ data and five training and five external test sets for IC_50_ data, including both quantitative and qualitative descriptions, were created for each antitarget.

**Table 1:** Data related with antitargets and the number of compounds with Ki and IC50 values in data sets.

Target	UniProt ID	Chembl Target ID	K_i_	IC_50_	Effects at antagonism or inhibition
Acetylcholinesterase	P22303	CHEMBL220	272	2573	↓ BP; ↓ HR; ↑ GI motility (↓ at high doses); bronchoconstriction; ↑ respiratory secretions; anaphylaxis; anorexiant; arrhythmogenic; asystole; colic; diarrhea; emetic; gastrointestinal hemorrhage; headache; hypotension; muscle weakness; nausea; neurotoxic; nightmare; respiratory failure; sialorrhea; sweating; ulcer, gastric; urticaria
Adenosine receptor A2a	P29274	CHEMBL251	3258	213	Platelet aggregation; ↑ BP; nervousness (tremors, agitation); arousal; insomnia
Alpha-1A adrenergic receptor	P35348	CHEMBL229	942	100	↓ smooth muscle tone; orthostatic hypotension and ↑ HR; dizziness; impact on various aspects of sexual function; flushing; hypotension; impotence; nasal congestion; postural (orthostatic) hypotension; tachycardia; weakness
Alpha-2A adrenergic receptor	P08913	CHEMBL1867	557	201	↑ GI motility; ↑ insulin secretion; hypertension exacerbates heart failure; anxiety; depression
Beta-1 adrenergic receptor	P08588	CHEMBL213	278	512	↓ BP; ↓ HR; ↓ cardiac output; cardiotoxicity; heart failure
Beta-2 adrenergic receptor	P07550	CHEMBL210	352	472	↓ BP; increased bronchospasm
Androgen receptor	P10275	CHEMBL1871	631	1054	↓ spermatogenesis; impotence; gynecomastia, mastodynia; ↑ in breast carcinoma
Muscarinic acetylcholine receptor M1	P11229	CHEMBL216	635	544	↓ cognitive function; ↓ gastric acid secretion; blurred vision
Muscarinic acetylcholine receptor M2	P08172	CHEMBL211	799	422	Tachycardia; bronchoconstriction; tremors
Muscarinic acetylcholine receptor M3	P20309	CHEMBL245	644	606	Constipation; blurred vision; pupil dilation; dry mouth
Cannabinoid receptor 1	P21554	CHEMBL218	1998	904	↑ weight loss; emesis; depression
Cannabinoid receptor 2	P34972	CHEMBL253	2375	592	↑ inflammation; ↓ bone mass
D(1A) dopamine receptor	P21728	CHEMBL2056	681	106	Dyskinesia; parkinsonian symptoms (tremors); anti-emetic effects; depression; anxiety; suicidal intent
D(2) dopamine receptor	P14416	CHEMBL217	3946	431	Orthostatic hypotension; drowsiness; ↑ GI motility; dyskinesia; extrapyramidal effect; sedative
Endothelin-1 receptor	P25101	CHEMBL252	155	894	Teratogenicity
Histamine H1 receptor	P35367	CHEMBL231	753	264	Sedation; ↓ allergic responses; ↑ body weight; dizziness; extrapyramidal effect; hypnotic; hypotension; lassitude; tinnitus; xerostomia
5-hydroxytryptamine receptor 1A	P08908	CHEMBL214	2505	432	Anxiogenic
5-hydroxytryptamine receptor 1B	P28222	CHEMBL1898	662	266	↑ aggression
5-hydroxytryptamine receptor 2A	P28223	CHEMBL224	1768	659	hypnotic; sedative
5-hydroxytryptamine receptor 2B	P41595	CHEMBL1833	705	248	Possible cardiac effects, especially during embryonic development
Potassium voltage-gated channel subfamily H member 2	Q12809	CHEMBL240	935	4078	Prolongation of QT interval of ECG
Tyrosine-protein kinase Lck	P06239	CHEMBL258	364	1322	T cell inhibition; SCID-like immunodeficiency
Amine oxidase [flavin-containing] A	P21397	CHEMBL1951	342	1031	↑ BP when combined with amines such as tyramine; drug–drug interaction potential; dizziness; sleep disturbances; nausea
Neuropeptide Y receptor type 1	P25929	CHEMBL4777	321	304	Anxiogenic
Glucocorticoid receptor	P04150	CHEMBL2034	632	1086	Hypoglycemia
Delta-type opioid receptor	P41143	CHEMBL236	1603	534	↑ BP; ↑ cardiac contractility
Mu-type opioid receptor	P35372	CHEMBL233	1816	663	↑ GI motility; dyspepsia; flatulence
Sodium-dependent noradrenaline transporter	P23975	CHEMBL222	1346	1371	↑ HR; ↑ BP; ↑ locomotor activity; constipation; abuse potential
Sodium-dependent dopamine transporter	Q01959	SLC6A3	1195	1183	Addictive psychostimulation; dopaminergic hyperactivity; depression; parkinsonism; attention deficit–hyperactivity disorder; psychotic disorders; seizures; dystonia; dyskinesia; acne
Sodium-dependent serotonin transporter	P31645	CHEMBL228	1868	1938	↑ GI motility; ↓ upper GI transit; ↓ plasma renin; ↑ other serotonin-mediated effects; insomnia; anxiety; nausea; sexual dysfunction

*BP, blood pressure; ECG, electrocardiogram; GI, gastrointestinal; HR, heart rate; SCID, severe-combined immunodeficiency.*

### GUSAR Software

The (Q)SAR models were created by GUSAR software^2^, which used quantitative neighbourhoods of atoms (QNA), multilevel neighbourhoods of atom (MNA), and whole-molecule descriptors with self-consistent regression (Lagunin et al., 2007; Filimonov et al., 2009; Lagunin et al., 2011). QNA descriptors are calculated by two functions, P and Q. The values for P and Q for each atom *i* are calculated as:

$$P_{i}=B_{i}\sum_{k}{\left(\mathrm{Exp}(-\frac{1}{2}C)\right)}_{\mathrm{ik}}B_{k},$$

$$Q_{i}=B_{i}\sum_{k}{\left(\mathrm{Exp}(-\frac{1}{2}C)\right)}_{\mathrm{ik}}B_{k}A_{k},$$

where *k* is all other atoms in the molecule and

$$A_{\text{k}}=\frac{1}{2}({\mathrm{IP}}_{\text{k}}+{\mathrm{EA}}_{\text{k}}),B_{\text{k}}={\left({\mathrm{IP}}_{\text{k}}-{\mathrm{EA}}_{\text{k}}\right)}^{-\frac{1}{2}}$$

Here, IP is the ionization potential, EA is the electron affinity for each atom, and C is the connectivity matrix for the molecule. QNA descriptors describe each particular atom of a molecule; at the same time, each *P* or *Q* value depends on the total molecule composition and structure. Two-dimensional Chebyshev polynomials are used for approximating the functions P and Q over all atoms of the molecule. A detailed description of QNA descriptors is represented in the publication of Filimonov et al. (2009).

MNA descriptors (Filimonov et al., 1999) are based on the molecular structure representation, which includes hydrogens according to the valences and partial charges of other atoms and does not specify the types of bonds. MNA descriptors are generated as a recursively defined sequence:

• zero-level MNA descriptor for each atom is the mark *A* of the atom itself;
• any next-level MNA descriptor for the atom is the sub-structure notation *A* (*D*_1_*D*_2_...*D*_i_...),

where *D*_i_ is the previous-level MNA descriptor for i–th immediate neighbor of the atom A.

The mark of the atom may include not only the atomic type but also any additional information about the atom. In particular, if the atom is not included in the ring, it is marked by “-”. The neighbor descriptors *D*_1_*D*_2_...*D*_i_... are arranged in a unique manner, for example, in lexicographic order. The iterative process of MNA descriptors generation can be continued covering first, second, and so on, neighborhoods of each atom.

For regression analysis, this molecule structure representation was transformed using the original PASS (Prediction of Activity Spectra for Substances) algorithm (Lagunin et al., 2011). This algorithm estimates the biological activity profiles for chemical compounds using MNA descriptors as input parameters. Therefore, we used the results of PASS prediction as independent variables for regression analysis. The results of PASS prediction are given as a list of biological activities, for which the difference between probabilities of being active (Pa) and inactive (Pi) was calculated. The activities from the list of predicted biological activities were randomly selected as input independent variables for regression analysis. This allows obtaining different QSAR models. GUSAR incorporates a PASS version that predicts 4130 types of biological activity. This version of PASS has a mean prediction accuracy of approximately 95% calculated by leave-one-out cross-validation procedure (Filimonov et al., 2014). The list of predictable biological activities currently includes 501 pharmacotherapeutic effects (e.g., antihypertensive, hepatoprotectant, and nootropic), 3295 mechanisms of action (e.g., 5-hydroxytryptamine antagonist, acetylcholine M1 receptor agonist, and cyclooxygenase inhibitor), 57 adverse and toxic effects (e.g., carcinogenic, mutagenic, and hematotoxic), 199 metabolic terms (e.g., CYP1A inducer, CYP1A1 inhibitor, and CYP3A4 substrate), 49 transporter proteins (e.g., P-glycoprotein 3 inhibitor, nucleoside transporters inhibitors, and proline transporter inhibitor), and 29 activities related to gene expression (e.g., TH expression enhancer, TNF expression inhibitor, and VEGF expression inhibitor). Therefore, the maximum number of independent variables for the creation of MNA models is 4130. The detailed description of realization of PASS in GUSAR is represented in the publication of Lagunin et al. (2011).

QNA and MNA descriptors do not provide information on the shape and volume of a molecule, although this information may be important for determination of structure-activity relationships. Therefore, these parameters, which are called whole-molecule descriptors, are also used in GUSAR. The whole-molecule descriptors used in GUSAR are: topological length, topological volume, lipophilicity, number of positive charges, number of negative charges, number of hydrogen bond acceptors, number of aromatic atoms, molecular weight, and number of halogen atoms. GUSAR uses estimation of the applicability domain based on different types of structural similarity using calculation of QNA and MNA descriptors (Zakharov et al., 2016).

GUSAR may provide an equation of any single (Q)SAR model (Lagunin et al., 2011). But because we used consensus (Q)SAR models from dozens or even hundreds of single (Q)SAR models, it is not possible to provide a general equation describing all selected variables. By this reason, the created consensus (Q)SAR models could not provide information about positive and negatively influencing descriptors. Instead that GUSAR shows positive and negative impact of each atom of the structure in the predicted value (Khayrullina et al., 2015). Analysis of the influence of atoms on the predicted value and the search for general relationships between the structures of active compounds interacting with antitargets is a separate task (because of each structure in the set should be analyzed), and it is beyond the scope of this publication.

### Evaluation of Prediction Accuracy

The following statistical parameters were calculated for estimating the accuracy of prediction:

(1) Sensitivity (Sens):
  Sensitivity = $\frac{\mathrm{TP}}{\mathrm{FN}+\mathrm{TP}}$, where TP is true positive, and FN is false negative numbers.
(2) Specificity (Spec):
  Specificity = $\frac{\mathrm{TN}}{\mathrm{TN}+\mathrm{FP}}$, where TN is true negative, and FP is false positive numbers.
(3) Accuracy:
  $$\mathrm{Accuracy}=\frac{\mathrm{TP}+\mathrm{TN}}{\mathrm{TP}+\mathrm{TN}+\mathrm{FP}+\mathrm{FN}}$$
(4) Balanced accuracy (BA): balance between sensitivity and specificity:
  $$\mathrm{BA}=\frac{\mathrm{Sensitivity}+\mathrm{Specificity}}{2}$$
(5) Root mean square error (RMSE):
  $$\mathrm{RMSE}=\sqrt{\frac{\sum {\left(y_{\exp}-y_{\mathrm{pred}}\right)}^{2}}{n}}$$
(6) *R*-squared, coefficient of determination:
  $$R^{2}=1-\frac{\sum {\left(y_{\exp}-y_{\mathrm{pred}}\right)}^{2}}{\sum {\left(y_{\exp}-y_{\mathrm{mean}}\right)}^{2}},$$

where *y*_exp_ – experimental value, *y*_pred_ – predicted value, and *y*_mean_ – average value of experimental values in a training set.

### Y-Randomization Procedure

Y-Randomization procedure is included in GUSAR software and allows to be ensuring that the developed continues QSAR models are robust and do not have the over fitting (Wold and Eriksson, 1995). In this procedure, the dependent-variable vector, Y vector (K_i_ or IC_50_ values in our case), is randomly shuffled and a new QSAR model is developed using the original independent variable matrix. It is expected that the resulting models should generally have low *Q*^2^ values. This procedure was repeated five times for each model, and then the average *Q*^2^ value was calculated.

## Results and Discussion

Three hundred twenty SAR and 320 QSAR models with modified calculation of descriptors and regression coefficients were created by GUSAR software for each from five training sets (five training sets with qualitative and quantitative data for K_i_ or IC_50_ values for each target) with internal validation (five times 20% from the training set was randomly used as an internal test set; this procedure is included into GUSAR). As a result, one consensus SAR model and one consensus QSAR model were created for each training set based on the appropriate single (Q)SAR model with *R*^2^_train_ and Q^2^_train_ and average *R*^2^ calculated for internal validation sets more than 0.5. If *R*^2^ of internal validation for (Q)SAR model was less than 0.5, then the model was excluded from the final consensus model [excluding QSAR models for D(1A) and D(2) dopamine receptors, histamine H1 and 5-hydroxytryptamine 2B receptors created on the basis of IC_50_ data]. The final predicted values for tested compounds were calculated using a weighted average of the predictions from the obtained (Q)SAR models. Each model is based on a different set of descriptors, and its predictions for each compound were weighted according to the similarity value that was calculated during the applicability domain assessment.

After SAR and QSAR consensus models were created based on a training set, they were used for prediction of inhibition of the antitarget by compounds from the appropriate external test set. It was repeated for five training sets with K_i_ values and five training sets with IC_50_ values for each antitarget (fivefold CV procedure). The average characteristics of the created (Q)SAR models including average results of Y-randomization procedure (Q^2^_Y–rand_) are represented in **Supplementary Tables S1, S2**. It was appeared that all Q^2^_Y–rand_ values for all QSAR models were less 0.15. The average Q^2^_Y–rand_ values were from 0.026 to 0.06 and from 0.026 to 0.078 for QSAR models created based on K_i_ and IC_50_ data, respectively. It is significant less in comparison with Q^2^ values calculated based on original data of the training sets and displays robustness of the given models.

**FIGURE 1:**
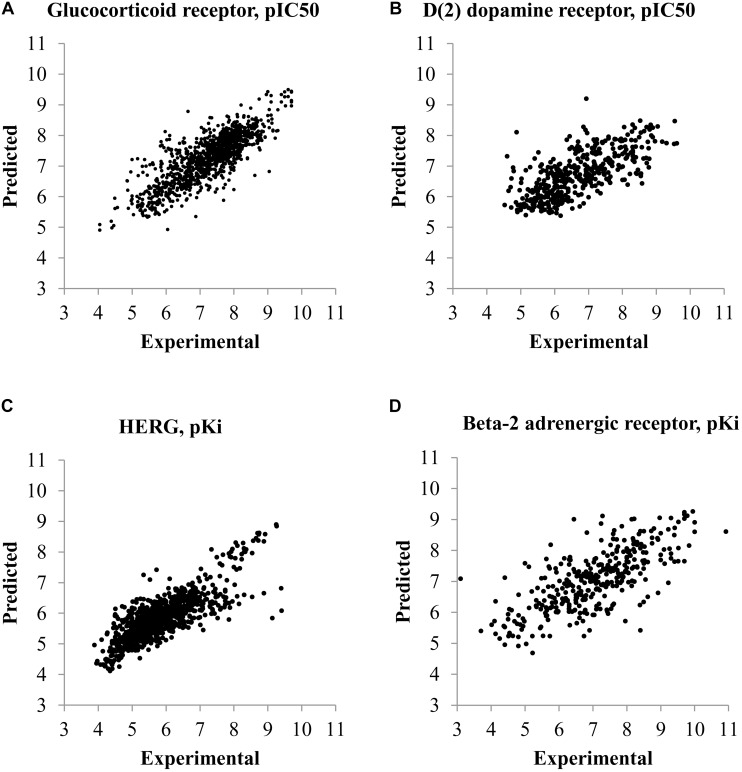
Plots of predicted and experimental values for the best and worst QSAR models by RMSE values calculated during fivefold cross-validation procedure. **(A)** QSAR model for prediction of pIC_50_ values of compounds interacting with glucocorticoid receptor (the best QSAR model for IC_50_ values). **(B)** QSAR model for prediction of pIC_50_ values of compounds interacting with D(2) dopamine receptor (the worst QSAR model for IC_50_ values). **(C)** QSAR model for prediction of pK_i_ values of compounds interacting with HERG channel (Potassium voltage-gated channel subfamily H member 2) (the best QSAR model for K_i_ values). **(D)** QSAR model for prediction of pK_i_ values of compounds interacting with Beta-2 adrenergic receptor (the worst QSAR model for K_i_ values).

The plots between predicted and experimental values for the best and worst QSAR models by RMSE values calculated by fivefold cross-validation are displayed in **Figure 1**. The relations between predicted and experimental values for others QSAR models are within these extreme cases.

**FIGURE 2:**
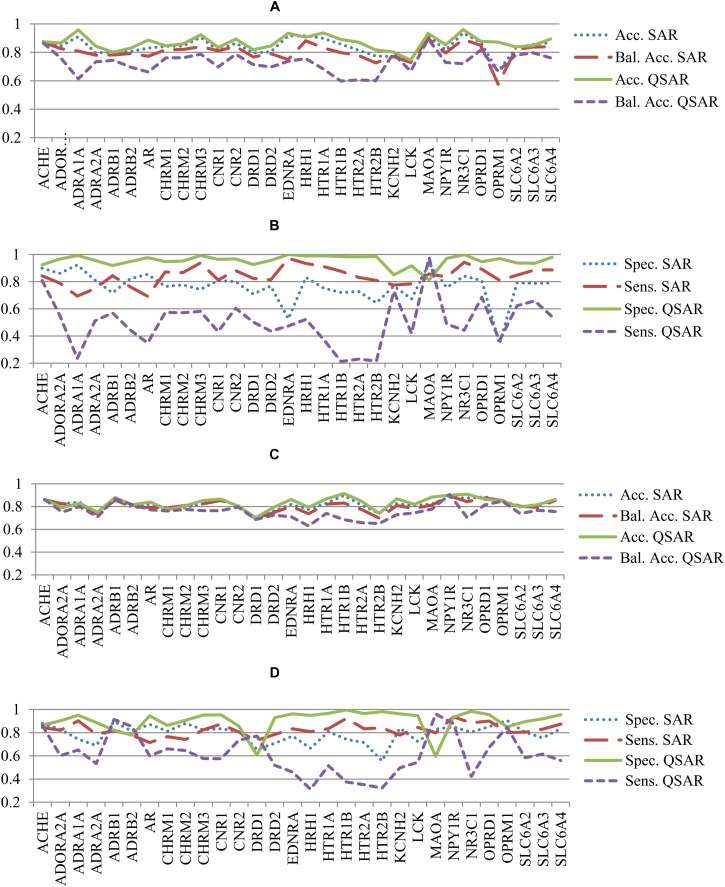
Comparison of parameters of accuracy of prediction for SAR and QSAR models calculated by the fivefold cross-validation procedure for all antitargets. **(A)** Comparison of *Accuracy* (Acc.) and *Balanced Accuracy* (Bal. Acc.) between SAR and QSAR models for K_i_ data. **(B)** Comparison of *Sensitivity* (Sens.) and *Specificity* (Spec.) between SAR and QSAR models for K_i_ data. **(C)** Comparison of *Accuracy* (Acc.) and *Balanced Accuracy* (Bal. Acc.) between SAR and QSAR models for IC_50_ data. **(D)** Comparison of *Sensitivity* (Sens.) and *Specificity* (Spec.) between SAR and QSAR models for IC_50_ data.

**FIGURE 3:**
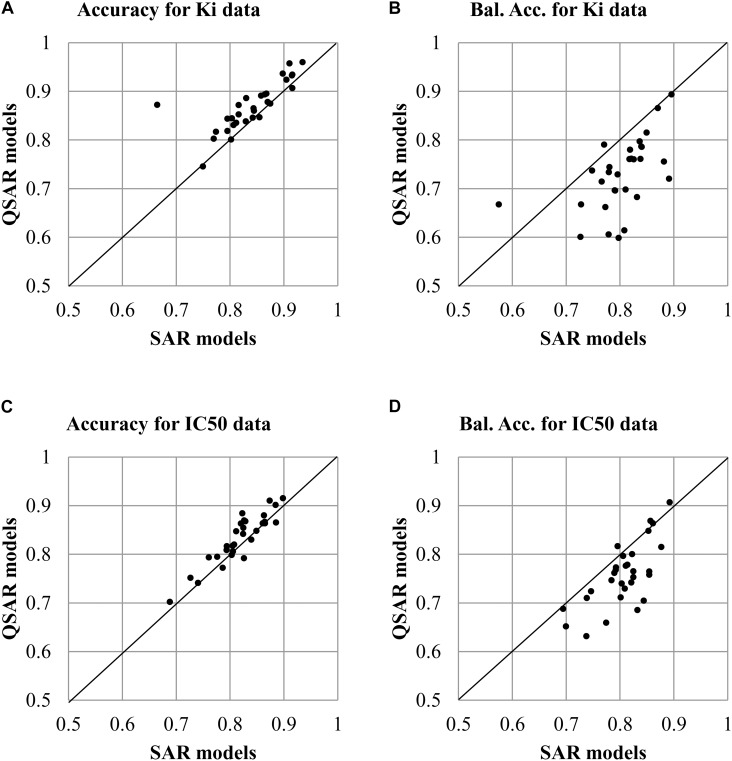
Correlation of accuracy of prediction between SAR and QSAR models for all antitargets. **(A)** Correlation of *Accuracy* between SAR and QSAR models for K_i_ data. **(B)** Correlation of *Balanced Accuracy* (BA) between SAR and QSAR models for K_i_ data. **(C)** Correlation of *Accuracy* between SAR and QSAR models for IC_50_ data. **(D)** Correlation of *Balanced Accuracy* (BA) between SAR and QSAR models for IC_50_ data.

**FIGURE 4:**
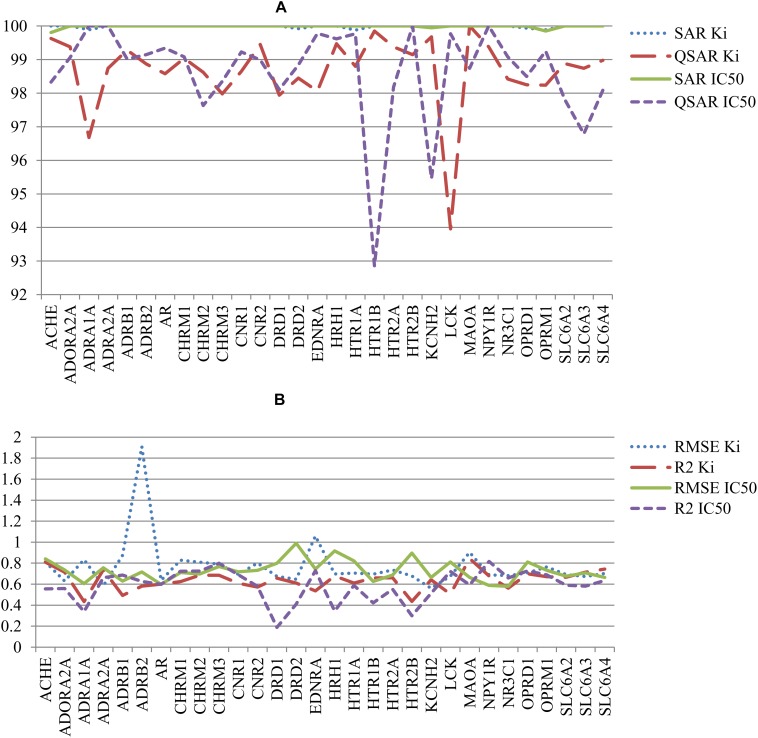
Comparison of quality of (Q)SAR models for K_i_ and IC_50_ data for all antitargets. **(A)** Comparison of the percent of compounds in applicability domain (AD) for SAR and QSAR models; **(B)** Comparison of *R*^2^ and RMSE values of QSAR models.

The statistical parameters describing accuracy of prediction and mentioned in the section “Materials and Methods” were calculated based on the prediction results given during the fivefold CV procedure for both SAR and QSAR models. To compare the accuracy of prediction of QSAR and SAR models, the quantitative results of prediction were transformed into qualitative ones according to the threshold mentioned in the section “Materials and Methods.” Statistical parameters of accuracy of prediction for SAR and QSAR models created based on K_i_ and IC_50_ data for all antitargets are represented in **Supplementary Tables S3, S4**, respectively. The graphical representation of statistical parameters of accuracy and their comparison are represented in **Figures 2–4**.

**Figures 2A,B** show a comparison of the accuracy between SAR and QSAR models created based on K_i_ values. **Figures 2C,D** show the results given based on IC_50_ values. The accuracy of the QSAR models was higher in most cases than the accuracy of SAR models for both K_i_ and IC_50_ values (**Figures 2A**, **1C**). The mean accuracy of prediction for K_i_ values was 0.84 and 0.87 for SAR and QSAR models, respectively. This is statistically significant difference (*p* < 0.05). The mean accuracy of prediction for IC_50_ values was 0.82 and 0.83 for SAR and QSAR models, respectively. This is statistically insignificant difference (*p* = 0.285). The reverse result was observed for balanced accuracy (SAR models: K_i_ data – 0.80, IC_50_ data – 0.81; QSAR models: K_i_ data – 0.73, IC_50_ data – 0.76). The difference in balanced accuracy between SAR and QSAR models is statistically significant in both cases, for K_i_ and for IC_50_ values (*p* < 0.05). Specificity and sensitivity were similar for SAR and QSAR models (**Figures 2B**, **1D**). The mean value of specificity was higher for QSAR models for both K_i_ and IC_50_ data (SAR models: K_i_ data – 0.76, IC_50_ data – 0.79; QSAR models: K_i_ data – 0.95, IC_50_ data – 0.90). The mean value of sensitivity was higher for SAR models for both K_i_ and IC_50_ data (SAR models: K_i_ data – 0.84, IC_50_ data – 0.82; QSAR models: K_i_ data – 0.50, IC_50_ data – 0.61).

The analysis of values of accuracy and balanced accuracy of SAR and QSAR models (**Supplementary Tables S1, S2**) shows that there is a correlation between them. **Figures 3A,B** show a correlation between accuracy and balanced accuracy for both SAR and QSAR models created based on K_i_ data. **Figures 3C,D** show a correlation between accuracy and balanced accuracy for SAR and QSAR models created based on IC_50_ data. One may see that in the both cases, the correlation between accuracy of SAR and QSAR models was higher than for balanced accuracy (**Figure 3**). If the values correlate, it means that there is no preference between SAR and QSAR models for the appropriate criterion of accuracy. But similar accuracy is achieved by different ways in the most cases (high sensitivity or high specificity, see **Figures 2B,D**). One can decide what is more important in the study: find as many as possible active compounds (the models with highest sensitivity should be selected) or reduce the number of false positive prediction (the models with highest specificity should be selected). The absence of correlation between the studied parameters shows that one of methods has preference. The values above the line show that QSAR models better than SAR ones. The values below the line show that SAR models better than QSAR ones. All cases excluding one which is displayed in **Figure 3C** (Correlation of *Accuracy* between SAR and QSAR models for IC_50_ data) had statistically significant difference between the values of SAR and QSAR models (*p* < 0.05). The values of balanced accuracy is the most important criterion for estimation of accuracy of prediction because of many used datasets were unbalanced (the number of active and inactive compounds is significant different). Therefore, the given results showed that SAR models are the more preferable for the use of prediction of drug adverse reactions.

The other parameters of SAR and QSAR models are represented in **Figure 4**. **Figure 4A** shows the percent of compounds in applicability domain (AD) of SAR and QSAR models. The number of compounds in AD was 100% approximately for all SAR models. At the same time, the number of compounds in AD approximately for all QSAR models was less 100%. The mean value of percent of compound in AD for SAR and QSAR models was 99.9% and 98.6%, respectively. The highest present of compounds in applicability domain displays advantage and better predictive power for SAR models in comparison with QSAR models. **Figure 4B** shows the comparison of RMSE and *R*^2^ values for QSAR models created on K_i_ and IC_50_ data. Clear features of distribution of these characteristics cannot be seen, but in general, the mean value of *R*^2^ for QSAR models based on K_i_ data was higher than one for IC_50_ data (0.64 and 0.57, respectively). The mean RMSE value for QSAR models based on IC_50_ data was less than one for K_i_ data (0.73 and 0.77, respectively). However, if we delete the RMSE value for the QSAR model created based on K_i_ data for the beta-2 adrenergic receptor, the mean RMSE value also became 0.73 for the other QSAR models created based on K_i_ data. It means that both K_i_ and IC_50_ values can be reliably used to predict interactions with antitargets. We may compare (Q)SAR models based on K_i_ and IC_50_ values only in general view because of they were created on different number of compounds and different structures. Nevertheless, we may reveal some features of the created models. The plots with comparison of Specificity and Sensitivity of (Q)SAR models created based on K_i_ and IC_50_ data are shown on **Supplementary Figure S1**. These plots display that SAR models based on IC_50_ values have Specificity better than SAR models based on K_i_ data for approximately half of antitargets. The biggest difference is shown for Mu-type opioid receptor (0.34 for K_i_ data and 0.97 for IC_50_ data). SAR models based on K_i_ data for others antitargets have better values of Specificity. The same picture we can see for Sensitivity of SAR models. Analysis of QSAR models revealed that majority of QSAR models based on K_i_ data had better Specificity value, whereas majority of QSAR models based on IC_50_ data had better Sensitivity value. High value of Sensitivity is more important for revealing possible adverse drug reaction than high value of Sensitivity. Analysis of Accuracy and Balanced Accuracy of (Q)SAR based on IC_50_ and K_i_ data (**Supplementary Figure S2**) show that the most (Q)SAR models based on K_i_ values have better values, whereas the values of Balanced Accuracy are higher at the most of QSAR models based on IC_50_ values.

## Conclusion

The creation of SAR and QSAR models based on the same data of compounds tested as inhibitors of 30 antitargets revealed some features related to the use of qualitative and quantitative data. They are valid to (Q)SAR models related to both K_i_ and IC_50_ values. SAR models tended to have more balanced prediction results when specificity and sensitivity have the closest values in comparison with QSAR models (**Figure 2**). High values of specificity and low values of sensitivity in QSAR models may be explained by the fact that at the given *R*^2^ values (0.64 and 0.59), prediction results tended to lie closer to the average values of K_i_ or IC_50_ in the training set. If a threshold of 1 μM divided the training set into different proportions of active and inactive compounds, then a difference between specificity and sensitivity may occur. At the same time, despite the difference of specificity and sensitivity between SAR and QSAR models, the values of accuracy and balanced accuracy for SAR correlated with those of QSAR models (**Figure 3**). This indicated that the prediction results of SAR and QSAR models would complement each other and that the use of both approaches would improve the quality of assessment of interaction between ligands and antitargets.

Another conclusion is that SAR models had advantages in the applicability domain. It may be related to the fact that the use ofqualitative data gives SAR models less sensitivity to experimental errors in K_i_ and IC_50_ values.

In this study, we also displayed that the modern experimental data and methods of (Q)SAR modeling allow for the creation of rather reasonable (Q)SAR models for prediction of interaction between compounds and dozens of antitargets. The used approaches may be applied to the creation of *in silico* panels for estimation of “ligand-antitarget” interactions during the drug design process.

## Author Contributions

AL designed the study, performed the data analysis, and wrote the manuscript with inputs of all authors. MR, AZ, NK, and BS created and validated (Q)SAR models. PP and SI created datasets and data analysis. DF and VP designed the study, analyzed the results, and wrote the manuscript.

## Conflict of Interest Statement

The authors declare that the research was conducted in the absence of any commercial or financial relationships that could be construed as a potential conflict of interest.

## Footnotes

1 http://www.way2drug.com/gusar/antitargets.html

2 http://www.way2drug.com/gusar/index.html
