# Supplementary material for: Comparison of Quantitative and Qualitative (Q)SAR Models Created for the Prediction of Ki and IC50 Values of Antitarget Inhibitors
Source: Front Pharmacol. 2018 Oct 10;9:1136. doi: 10.3389/fphar.2018.01136 (PMC6192375; doi:10.3389/fphar.2018.01136)

Supplementary Material

Comparison of quantitative and qualitative (Q)SAR models created for the prediction of K_i and IC_{50} values of antitarget inhibitors

Alexey A. Lagunin*, Maria A. Romanova, Anton D. Zadorozhny, Natalia S. Kurilenko, Boris V. Shilov, Pavel V. Pogodin, Sergey M. Ivanov, Dmitry A. Filimonov, Vladimir V. Poroikov*

*** Correspondence:** Alexey A. Lagunin: alexey.lagunin@ibmc.msk.ru
Vladimir V. Poroikov: vladimir.poroikov@ibmc.msk.ru

Figure S1. Comparison of Specificity and Sensitivity of (Q)SAR models created based on K_i and IC_{50} data

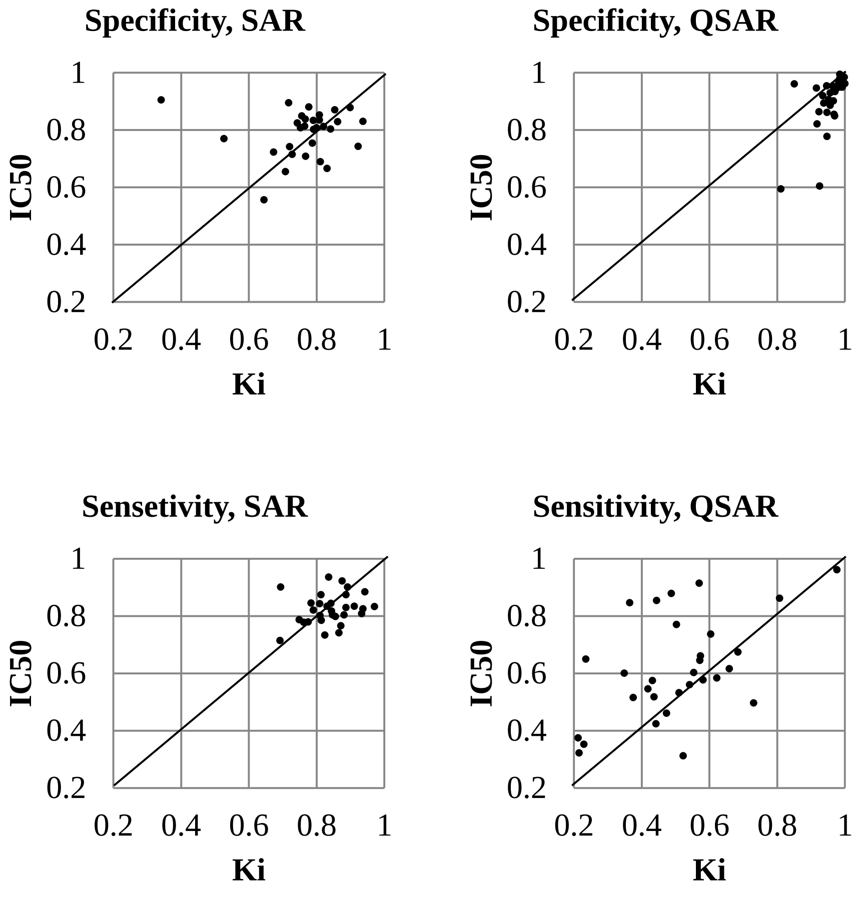

Supplement: Supplementary file 1 [file Data_Sheet_1.PDF]
