# Supplementary material for: Comparison of Quantitative and Qualitative (Q)SAR Models Created for the Prediction of Ki and IC50 Values of Antitarget Inhibitors
Source: Front Pharmacol. 2018 Oct 10;9:1136. doi: 10.3389/fphar.2018.01136 (PMC6192375; doi:10.3389/fphar.2018.01136)
Supplement: Supplementary file 4 [file Table_1.DOCX]

Supplementary Material

Comparison of quantitative and qualitative (Q)SAR models created for the prediction of K_i_ and IC_50_ values of antitarget inhibitors

Alexey A. Lagunin*, Maria A. Romanova, Anton D. Zadorozhny, Natalia S. Kurilenko, Boris V. Shilov, Pavel V. Pogodin, Sergey M. Ivanov, Dmitry A. Filimonov, Vladimir V. Poroikov*

*** Correspondence:** Alexey A. Lagunin: alexey.lagunin@ibmc.msk.ru
Vladimir V. Poroikov: vladimir.poroikov@ibmc.msk.ru

**Table S1.** Average characteristics of SAR and QSAR models created based on K_i_ data

| **Target** | **Gene** | **UniProt ID** | **SAR models** | | | | **QSAR models** | | | |
| --- | --- | --- | --- | --- | --- | --- | --- | --- | --- | --- |
|  |  |  | **Sens** | **Spec** | **BA** | **G-mean** | **R^2^** | **Q^2^** | **SD** | **Q^2^_Y-rand_** |
| Acetylcholinesterase | ACHE | P22303 | 0.881 | 0.906 | 0.894 | 0.893 | 0.993 | 0.823 | 0.785 | 0.047 |
| Adenosine receptor A2a | ADORA2A | P29274 | 0.819 | 0.877 | 0.848 | 0.848 | 0.991 | 0.740 | 0.605 | 0.026 |
| Alpha-1A adrenergic receptor | ADRA1A | P35348 | 0.901 | 0.893 | 0.897 | 0.897 | 0.936 | 0.593 | 0.737 | 0.038 |
| Alpha-2A adrenergic receptor | ADRA2A | P08913 | 0.790 | 0.869 | 0.830 | 0.828 | 0.957 | 0.720 | 0.616 | 0.053 |
| Beta-1 adrenergic receptor | ADRB1 | P08588 | 0.918 | 0.698 | 0.808 | 0.800 | 0.726 | 0.630 | 0.757 | 0.054 |
| Beta-2 adrenergic receptor | ADRB2 | P07550 | 0.802 | 0.892 | 0.847 | 0.846 | 0.757 | 0.626 | 0.855 | 0.053 |
| Androgen receptor | AR | P10275 | 0.818 | 0.822 | 0.820 | 0.819 | 0.953 | 0.649 | 0.600 | 0.042 |
| Muscarinic acetylcholine receptor M1 | CHRM1 | P11229 | 0.813 | 0.879 | 0.846 | 0.845 | 0.952 | 0.645 | 0.819 | 0.060 |
| Muscarinic acetylcholine receptor M2 | CHRM2 | P08172 | 0.838 | 0.873 | 0.855 | 0.855 | 0.954 | 0.699 | 0.804 | 0.041 |
| Muscarinic acetylcholine receptor M3 | CHRM3 | P20309 | 0.838 | 0.918 | 0.878 | 0.877 | 0.953 | 0.691 | 0.783 | 0.046 |
| Cannabinoid receptor 1 | CNR1 | P21554 | 0.882 | 0.794 | 0.838 | 0.837 | 0.968 | 0.669 | 0.645 | 0.038 |
| Cannabinoid receptor 2 | CNR2 | P34972 | 0.820 | 0.896 | 0.858 | 0.857 | 0.981 | 0.639 | 0.738 | 0.037 |
| D(1A) dopamine receptor | DRD1 | P21728 | 0.844 | 0.829 | 0.836 | 0.836 | 0.906 | 0.662 | 0.677 | 0.046 |
| D(2) dopamine receptor | DRD2 | P14416 | 0.828 | 0.806 | 0.817 | 0.817 | 0.958 | 0.649 | 0.622 | 0.026 |
| Endothelin-1 receptor | EDNRA | P25101 | 0.935 | 0.921 | 0.928 | 0.927 | 0.958 | 0.650 | 0.935 | 0.040 |
| Histamine H1 receptor | HRH1 | P35367 | 0.849 | 0.824 | 0.836 | 0.836 | 0.979 | 0.727 | 0.651 | 0.049 |
| 5-hydroxytryptamine receptor 1A | HTR1A | P08908 | 0.940 | 0.849 | 0.894 | 0.893 | 0.953 | 0.651 | 0.685 | 0.056 |
| 5-hydroxytryptamine receptor 1B | HTR1B | P28222 | 0.880 | 0.833 | 0.861 | 0.860 | 0.981 | 0.722 | 0.640 | 0.047 |
| 5-hydroxytryptamine receptor 2A | HTR2A | P28223 | 0.825 | 0.829 | 0.827 | 0.827 | 0.974 | 0.719 | 0.669 | 0.036 |
| 5-hydroxytryptamine receptor 2B | HTR2B | P41595 | 0.831 | 0.818 | 0.824 | 0.823 | 0.636 | 0.544 | 0.628 | 0.053 |
| Potassium voltage-gated channel subfamily H member 2 | KCNH2 | Q12809 | 0.778 | 0.862 | 0.820 | 0.819 | 0.987 | 0.712 | 0.510 | 0.039 |
| Tyrosine-protein kinase Lck | LCK | P06239 | 0.752 | 0.851 | 0.802 | 0.798 | 1.000 | 0.624 | 0.597 | 0.051 |
| Amine oxidase [flavin-containing] A | MAOA | P21397 | 0.919 | 0.922 | 0.921 | 0.921 | 0.991 | 0.850 | 0.879 | 0.046 |
| Neuropeptide Y receptortype 1 | NPY1R | P25929 | 0.905 | 0.840 | 0.862 | 0.862 | 0.987 | 0.711 | 0.657 | 0.032 |
| Glucocorticoid receptor | NR3C1 | P04150 | 0.909 | 0.933 | 0.921 | 0.920 | 0.963 | 0.624 | 0.637 | 0.044 |
| Delta-type opioid receptor | OPRD1 | P41143 | 0.898 | 0.839 | 0.869 | 0.868 | 0.942 | 0.707 | 0.702 | 0.035 |
| Mu-type opioid receptor | OPRM1 | P35372 | 0.922 | 0.773 | 0.847 | 0.844 | 0.995 | 0.695 | 0.746 | 0.035 |
| Sodium-dependent noradrenaline transporter | SLC6A2 | P23975 | 0.893 | 0.802 | 0.848 | 0.847 | 0.943 | 0.698 | 0.667 | 0.041 |
| Sodium-dependent dopamine transporter | SLC6A3 | Q01959 | 0.934 | 0.859 | 0.897 | 0.896 | 0.964 | 0.758 | 0.626 | 0.039 |
| Sodium-dependent serotonin transporter | SLC6A4 | P31645 | 0.910 | 0.828 | 0.869 | 0.868 | 0.969 | 0.761 | 0.688 | 0.036 |

Spec – specificity; Sens – sensitivity; BA – balanced accuracy; G-mean – geometrically mean; SD – Standard Deviation; Q^2^_Y-rand_ – Q^2^ calculated based on training set by Y-randomization test.
