# Supplementary material for: Comparison of Quantitative and Qualitative (Q)SAR Models Created for the Prediction of Ki and IC50 Values of Antitarget Inhibitors
Source: Front Pharmacol. 2018 Oct 10;9:1136. doi: 10.3389/fphar.2018.01136 (PMC6192375; doi:10.3389/fphar.2018.01136)
Supplement: Supplementary file 5 [file Table_2.DOCX]

Supplementary Material

Comparison of quantitative and qualitative (Q)SAR models created for the prediction of K_i_ and IC_50_ values of antitarget inhibitors

Alexey A. Lagunin*, Maria A. Romanova, Anton D. Zadorozhny, Natalia S. Kurilenko, Boris V. Shilov, Pavel V. Pogodin, Sergey M. Ivanov, Dmitry A. Filimonov, Vladimir V. Poroikov*

*** Correspondence:** Alexey A. Lagunin: alexey.lagunin@ibmc.msk.ru
Vladimir V. Poroikov: vladimir.poroikov@ibmc.msk.ru

**Table S2.** Average characteristics of SAR and QSAR models created based on IC_50_ data

| **Target** | **Gene** | **UniProt ID** | **SAR models** | | | | **QSAR models** | | | |
| --- | --- | --- | --- | --- | --- | --- | --- | --- | --- | --- |
|  |  |  | **Sens** | **Spec** | **BA** | **G-mean** | **R^2^** | **Q^2^** | **SD** | **Q^2^_Y-rand_** |
| Acetylcholinesterase | ACHE | P22303 | 0.872 | 0.836 | 0.854 | 0.854 | 0.968 | 0.713 | 0.876 | 0.026 |
| Adenosine receptor A2a | ADORA2A | P29274 | 0.831 | 0.859 | 0.845 | 0.844 | 0.992 | 0.632 | 0.673 | 0.062 |
| Alpha-1A adrenergic receptor | ADRA1A | P35348 | 0.801 | 0.893 | 0.847 | 0.844 | 0.960 | 0.801 | 0.497 | 0.078 |
| Alpha-2A adrenergic receptor | ADRA2A | P08913 | 0.843 | 0.795 | 0.819 | 0.817 | 1.000 | 0.739 | 0.673 | 0.051 |
| Beta-1 adrenergic receptor | ADRB1 | P08588 | 0.954 | 0.807 | 0.880 | 0.877 | 0.986 | 0.755 | 0.556 | 0.039 |
| Beta-2 adrenergic receptor | ADRB2 | P07550 | 0.790 | 0.896 | 0.843 | 0.841 | 0.970 | 0.680 | 0.662 | 0.039 |
| Androgen receptor | AR | P10275 | 0.830 | 0.792 | 0.811 | 0.810 | 0.970 | 0.655 | 0.598 | 0.037 |
| Muscarinic acetylcholine receptor M1 | CHRM1 | P11229 | 0.832 | 0.787 | 0.809 | 0.808 | 0.984 | 0.747 | 0.700 | 0.048 |
| Muscarinic acetylcholine receptor M2 | CHRM2 | P08172 | 0.887 | 0.773 | 0.830 | 0.827 | 0.975 | 0.740 | 0.680 | 0.043 |
| Muscarinic acetylcholine receptor M3 | CHRM3 | P20309 | 0.863 | 0.852 | 0.857 | 0.857 | 0.954 | 0.727 | 0.736 | 0.044 |
| Cannabinoid receptor 1 | CNR1 | P21554 | 0.945 | 0.847 | 0.896 | 0.894 | 0.978 | 0.747 | 0.652 | 0.042 |
| Cannabinoid receptor 2 | CNR2 | P34972 | 0.854 | 0.818 | 0.836 | 0.835 | 0.965 | 0.675 | 0.653 | 0.044 |
| D(1A) dopamine receptor | DRD1 | P21728 | 0.750 | 0.706 | 0.728 | 0.725 | 0.475 | 0.343 | 0.792 | 0.062 |
| D(2) dopamine receptor | DRD2 | P14416 | 0.826 | 0.807 | 0.816 | 0.816 | 0.668 | 0.483 | 0.996 | 0.044 |
| Endothelin-1 receptor | EDNRA | P25101 | 0.864 | 0.813 | 0.839 | 0.838 | 0.994 | 0.743 | 0.726 | 0.035 |
| Histamine H1 receptor | HRH1 | P35367 | 0.875 | 0.805 | 0.840 | 0.839 | 0.601 | 0.483 | 0.827 | 0.046 |
| 5-hydroxytryptamine receptor 1A | HTR1A | P08908 | 0.907 | 0.830 | 0.868 | 0.867 | 0.990 | 0.685 | 0.724 | 0.049 |
| 5-hydroxytryptamine receptor 1B | HTR1B | P28222 | 0.864 | 0.911 | 0.888 | 0.887 | 0.923 | 0.618 | 0.517 | 0.043 |
| 5-hydroxytryptamine receptor 2A | HTR2A | P28223 | 0.852 | 0.805 | 0.828 | 0.828 | 0.996 | 0.638 | 0.620 | 0.046 |
| 5-hydroxytryptamine receptor 2B | HTR2B | P41595 | 0.730 | 0.849 | 0.789 | 0.785 | 0.568 | 0.436 | 0.832 | 0.061 |
| Potassium voltage-gated channel subfamily H member 2 | KCNH2 | Q12809 | 0.836 | 0.796 | 0.816 | 0.816 | 0.992 | 0.609 | 0.705 | 0.025 |
| Tyrosine-protein kinase Lck | LCK | P06239 | 0.785 | 0.845 | 0.815 | 0.815 | 0.997 | 0.754 | 0.762 | 0.036 |
| Amine oxidase [flavin-containing] A | MAOA | P21397 | 0.843 | 0.861 | 0.852 | 0.852 | 0.982 | 0.643 | 0.626 | 0.041 |
| Neuropeptide Y receptortype 1 | NPY1R | P25929 | 0.918 | 0.934 | 0.926 | 0.926 | 0.990 | 0.833 | 0.568 | 0.052 |
| Glucocorticoid receptor | NR3C1 | P04150 | 0.939 | 0.805 | 0.872 | 0.870 | 0.979 | 0.677 | 0.565 | 0.039 |
| Delta-type opioid receptor | OPRD1 | P41143 | 0.929 | 0.892 | 0.911 | 0.911 | 0.999 | 0.824 | 0.522 | 0.048 |
| Mu-type opioid receptor | OPRM1 | P35372 | 0.925 | 0.843 | 0.884 | 0.883 | 0.972 | 0.752 | 0.657 | 0.044 |
| Sodium-dependent noradrenaline transporter | SLC6A2 | P23975 | 0.841 | 0.797 | 0.819 | 0.818 | 0.911 | 0.601 | 0.675 | 0.048 |
| Sodium-dependent dopamine transporter | SLC6A3 | Q01959 | 0.833 | 0.802 | 0.817 | 0.817 | 0.856 | 0.584 | 0.719 | 0.052 |
| Sodium-dependent serotonin transporter | SLC6A4 | P31645 | 0.914 | 0.827 | 0.871 | 0.870 | 0.939 | 0.672 | 0.636 | 0.037 |

Spec – specificity; Sens – sensitivity; BA – balanced accuracy; G-mean – geometrically mean; SD – Standard Deviation; Q^2^_Y-rand_ – Q^2^ calculated based on training set by Y-randomization test.
