# Supplementary material for: Comparison of Quantitative and Qualitative (Q)SAR Models Created for the Prediction of Ki and IC50 Values of Antitarget Inhibitors
Source: Front Pharmacol. 2018 Oct 10;9:1136. doi: 10.3389/fphar.2018.01136 (PMC6192375; doi:10.3389/fphar.2018.01136)
Supplement: Supplementary file 6 [file Table_3.DOCX]

Supplementary Material

Comparison of quantitative and qualitative (Q)SAR models created for the prediction of K_i_ and IC_50_ values of antitarget inhibitors

Alexey A. Lagunin*, Maria A. Romanova, Anton D. Zadorozhny, Natalia S. Kurilenko, Boris V. Shilov, Pavel V. Pogodin, Sergey M. Ivanov, Dmitry A. Filimonov, Vladimir V. Poroikov*

*** Correspondence:** Alexey A. Lagunin: alexey.lagunin@ibmc.msk.ru
Vladimir V. Poroikov: vladimir.poroikov@ibmc.msk.ru

**Table S3.** Accuracy of prediction of SAR and QSAR models created based on of K_i_ data for test sets during 5-fold cross-validation

| **Target** | **Gene** | **UniProt ID** | **SAR** | | | | | **QSAR** | | | | | | |
| --- | --- | --- | --- | --- | --- | --- | --- | --- | --- | --- | --- | --- | --- | --- |
|  |  |  | **Spec** | **Sens** | **Acc** | **BA** | **AD** | **Speс** | **Sens** | **Acc** | **BA** | **AD** | **RMSE** | **R^2^** |
| Acetylcholinesterase | ACHE | P22303 | 0.90 | 0.84 | 0.88 | 0.87 | 100.00 | 0.92 | 0.81 | 0.87 | 0.87 | 99.63 | 0.81 | 0.81 |
| Adenosine receptor A2a | ADORA2A | P29274 | 0.86 | 0.79 | 0.84 | 0.83 | 100.00 | 0.97 | 0.55 | 0.87 | 0.76 | 99.39 | 0.63 | 0.71 |
| Alpha-1A adrenergic receptor | ADRA1A | P35348 | 0.92 | 0.69 | 0.91 | 0.81 | 99.89 | 0.99 | 0.24 | 0.96 | 0.61 | 96.68 | 0.83 | 0.43 |
| Alpha-2A adrenergic receptor | ADRA2A | P08913 | 0.81 | 0.75 | 0.80 | 0.78 | 100.00 | 0.96 | 0.51 | 0.84 | 0.73 | 98.74 | 0.58 | 0.74 |
| Beta-1 adrenergic receptor | ADRB1 | P08588 | 0.72 | 0.84 | 0.80 | 0.78 | 100.00 | 0.92 | 0.57 | 0.80 | 0.74 | 99.28 | 0.87 | 0.49 |
| Beta-2 adrenergic receptor | ADRB2 | P07550 | 0.82 | 0.76 | 0.81 | 0.79 | 100.00 | 0.95 | 0.44 | 0.83 | 0.70 | 98.87 | 1.91 | 0.58 |
| Androgen receptor | AR | P10275 | 0.85 | 0.69 | 0.83 | 0.77 | 100.00 | 0.98 | 0.35 | 0.89 | 0.66 | 98.58 | 0.64 | 0.60 |
| Muscarinic acetylcholine receptor M1 | CHRM1 | P11229 | 0.76 | 0.87 | 0.84 | 0.82 | 100.00 | 0.95 | 0.57 | 0.85 | 0.76 | 99.04 | 0.83 | 0.62 |
| Muscarinic acetylcholine receptor M2 | CHRM2 | P08172 | 0.78 | 0.87 | 0.84 | 0.82 | 100.00 | 0.95 | 0.57 | 0.86 | 0.76 | 98.62 | 0.81 | 0.69 |
| Muscarinic acetylcholine receptor M3 | CHRM3 | P20309 | 0.74 | 0.94 | 0.91 | 0.84 | 100.00 | 0.99 | 0.58 | 0.92 | 0.79 | 97.98 | 0.79 | 0.68 |
| Cannabinoid receptor 1 | CNR1 | P21554 | 0.81 | 0.81 | 0.81 | 0.81 | 100.00 | 0.96 | 0.43 | 0.84 | 0.70 | 98.65 | 0.69 | 0.61 |
| Cannabinoid receptor 2 | CNR2 | P34972 | 0.80 | 0.88 | 0.86 | 0.84 | 100.00 | 0.97 | 0.60 | 0.89 | 0.79 | 99.45 | 0.80 | 0.57 |
| D(1A) dopamine receptor | DRD1 | P21728 | 0.71 | 0.82 | 0.80 | 0.77 | 100.00 | 0.93 | 0.50 | 0.82 | 0.71 | 97.94 | 0.67 | 0.66 |
| D(2) dopamine receptor | DRD2 | P14416 | 0.77 | 0.81 | 0.80 | 0.79 | 99.92 | 0.96 | 0.44 | 0.84 | 0.70 | 98.45 | 0.65 | 0.61 |
| Endothelin-1 receptor | EDNRA | P25101 | 0.53 | 0.97 | 0.92 | 0.75 | 100.00 | 1.00 | 0.47 | 0.93 | 0.74 | 98.06 | 1.06 | 0.54 |
| Histamine H1 receptor | HRH1 | P35367 | 0.83 | 0.93 | 0.92 | 0.88 | 100.00 | 0.99 | 0.52 | 0.91 | 0.76 | 99.47 | 0.70 | 0.68 |
| 5-hydroxytryptamine receptor 1A | HTR1A | P08908 | 0.75 | 0.91 | 0.90 | 0.83 | 99.88 | 0.99 | 0.38 | 0.94 | 0.68 | 98.80 | 0.71 | 0.61 |
| 5-hydroxytryptamine receptor 1B | HTR1B | P28222 | 0.72 | 0.88 | 0.86 | 0.80 | 100.00 | 0.98 | 0.21 | 0.89 | 0.60 | 99.85 | 0.70 | 0.66 |
| 5-hydroxytryptamine receptor 2A | HTR2A | P28223 | 0.73 | 0.83 | 0.82 | 0.78 | 100.00 | 0.98 | 0.23 | 0.87 | 0.61 | 99.38 | 0.73 | 0.66 |
| 5-hydroxytryptamine receptor 2B | HTR2B | P41595 | 0.64 | 0.81 | 0.77 | 0.73 | 100.00 | 0.99 | 0.22 | 0.82 | 0.60 | 99.15 | 0.68 | 0.44 |
| Potassium voltage-gated channel subfamily H member 2 | KCNH2 | Q12809 | 0.77 | 0.77 | 0.77 | 0.77 | 100.00 | 0.85 | 0.73 | 0.80 | 0.79 | 99.68 | 0.56 | 0.64 |
| Tyrosine-protein kinase Lck | LCK | P06239 | 0.67 | 0.78 | 0.75 | 0.73 | 100.00 | 0.92 | 0.42 | 0.75 | 0.67 | 93.96 | 0.68 | 0.51 |
| Amine oxidase [flavin-containing] A | MAOA | P21397 | 0.94 | 0.86 | 0.92 | 0.90 | 100.00 | 0.81 | 0.98 | 0.93 | 0.89 | 100.00 | 0.90 | 0.84 |
| Neuropeptide Y receptor type 1 | NPY1R | P25929 | 0.76 | 0.84 | 0.82 | 0.80 | 100.00 | 0.97 | 0.49 | 0.85 | 0.73 | 99.38 | 0.69 | 0.68 |
| Glucocorticoid receptor | NR3C1 | P04150 | 0.84 | 0.94 | 0.94 | 0.89 | 100.00 | 1.00 | 0.44 | 0.96 | 0.72 | 98.42 | 0.68 | 0.56 |
| Delta-type opioid receptor | OPRD1 | P41143 | 0.81 | 0.89 | 0.87 | 0.85 | 99.94 | 0.95 | 0.68 | 0.88 | 0.82 | 98.25 | 0.71 | 0.70 |
| Mu-type opioid receptor | OPRM1 | P35372 | 0.34 | 0.81 | 0.67 | 0.58 | 99.89 | 0.97 | 0.36 | 0.87 | 0.67 | 98.24 | 0.76 | 0.67 |
| Sodium-dependent noradrenaline transporter | SLC6A2 | P23975 | 0.79 | 0.85 | 0.83 | 0.82 | 100.00 | 0.94 | 0.62 | 0.84 | 0.78 | 98.88 | 0.69 | 0.67 |
| Sodium-dependent dopamine transporter | SLC6A3 | Q01959 | 0.79 | 0.89 | 0.86 | 0.84 | 100.00 | 0.94 | 0.66 | 0.85 | 0.80 | 98.74 | 0.67 | 0.71 |
| Sodium-dependent serotonin transporter | SLC6A4 | P31645 | 0.79 | 0.89 | 0.87 | 0.84 | 100.00 | 0.98 | 0.54 | 0.90 | 0.76 | 98.98 | 0.70 | 0.74 |
| **Average** |  |  | **0.76** | **0.84** | **0.84** | **0.80** | **99.98** | **0.95** | **0.50** | **0.87** | **0.73** | **98.69** | **0.77** | **0.64** |

Spec – specificity; Sens – sensitivity; Acc – accuracy; BA – balanced accuracy; AD - % of compounds in applicability domain
